# Supplementary material for: Constitutive auxin response in Physcomitrella reveals complex interactions between Aux/IAA and ARF proteins
Source: eLife. 2016 Jun 1;5:e13325. doi: 10.7554/eLife.13325 (PMC4889330; doi:10.7554/eLife.13325)
Supplement: Supplementary file 3. — DOI: http://dx.doi.org/10.7554/eLife.13325.020 [file elife-13325-supp3.docx]

Supplementary File 3

| PML52 | CACCggatccCAGTCGCTTACTGTGAGGTTTCG |
| --- | --- |
| PML53 | AAGGATCCTATCTCTTCCCTAAGCTGCAGGCATG |
| PML59 | CACCactagtGGAGATTGCAATGGCGGGATGTGCG |
| PML60 | CCactagtcgtaaaatacttacaatcatgaaatccttccc |
| PML61 | CACCggatccggattgaagagtggcagtgggtg |
| PML62 | AAGGATCCtgaataatgtccaacttcactgctaaaag |
| PML63 | CACCggatccgagggtggtgcagtcaggtacaga |
| PML64 | AAGGATCCtgtctatgctgtacgctgcacaca |
| PML65 | CACCactagttgagaagcggatgcgtgtcgtatgag |
| PML66 | CCactagtgatcctctaccaactagatgaaactactgg |
| PML195 | TGGGGTTTCTACAGGACGTAAC |
| PML197 | cgccttcttgacgagttcttctg |
| PML267 | gatagtggaaaccgacgcccc |
| PML399 | CAGATGAGTCAGGAGCAACTTGAG |
| PML400 | ATTCGACGTGTTCACAGGAGACG |
| PML401 | AGATTGTCTGCCTTTGCTTGACC |
| PML402 | CATGCAGATCCTTAGCCACAAGC |
| PML405 | TGCGAGGAAGACGATGAATGGC |
| PML406 | GTTGTGCCGCAATCGCTTAGAC |
| PML409 | TTGCAGCAATTCTGAGGATGCC |
| PML419 | AAGGACAACGTGGAGGTGTTGG |
| PML455 | CACCATGCAATATTACGGAGGTATGCGAG |
| PML457 | GCCACTGCTCTGAACTGCAGAAAG |
| PML461 | CACCATGTACCAGTGCAGCGAGAGG |
| PML463 | TCGACCGCCAGAGTGTCCATG |
| PML506 | CACCGTGGAGAAAATGGTCAGCTCAG |
| PML507 | GTACCGGAGTACCTCCTCTCA |
| PML508 | TGAGAGGAGGTACTCCGGTAC |
| PML509 | CCCCAAAACCCCGGGGCACAAAGGTAGGTGGTCGGCG |
| PML510 | CCCCGGGGTTTTGGGGTGGTTGTCTAAACTTCAATTGGC |
| PML511 | AACAACTTGCACGGCTGCACC |
| PML512 | GGTGCAGCCGTGCAAGTTGTT |
| PML513 | TCCTCATTGGAGCAGCATGGG |
| PML551 | TCTCAGCAACGACGTGTCGAG |
| PML599 | caccGGATCCgcagcgttgttgcaacgttgg |
| PML600 | GGATCCcctctcttgcctgagcgccga |
| PML601 | CACCACTAGTAaggtgtctctcattttggatgtg |
| PML602 | ACTAGTacgctgacaaaccccacaatt |
| PML612 | TCCGCTCCTCACTGGAACGG |
| PML614 | TCAAACGGCCGAAACATTCTACG |
| PML615 | CAGCTCCGCTCCTTTCAGAATATG |
| PML618 | TCCCTCGTTTCATATGCTGGTTGC |
| PML619 | TCCTTCACGCTGCCTCTAATGAC |
| PML626 | TGGAGGTCACGGAATCACCATC |
| PML627 | ACACTGTCACTGCTATGCACAC |
| PML675 | CCAGTGAGCTAAGAGCATACATAGG |
| PML676 | ACGGTGTTAATTCCATAGGCTTTCA |
| PML677 | CACCGTCGACCGGAGACGAGTAGTAGGCTACTTTC |
| PML678 | GTCGACCTTTGCTTGCCGTTGTAGCCA |
| PML679 | CACCACTAGTGAATTAGTAGCCTATGGAGGTTTTAATGGA |
| PML680 | ACTAGTGGATCATTTGGTACCAACTAGAATTTTGATTC |
| PML703 | GGAATTTGTCTGTCGGAGGAAG |
| PML704 | TCCTACTGTGGGACACAACTAAG |
| PML749 | CTTTTAGCAGTGAAGTTGGACATTATTCA |
| PML750 | GCCTGCAGCTTAGGGAAGAGATA |
| PML751 | CATCCCGCCATTGCAATCTCC |
| PML753 | GTGTGCAGCGTACAGCATAGACA |
| PML754 | ATACGACACGCATCCGCTTCTCA |
| PML776 | CACCATGGCAGCTTCCAATCATTCAT |
| PML777 | TCTTGATCCCGCCATAGATGA |
| PML810 | TTGGAAAGACCGCCCAGCTATC |
| PML811 | GCTCCGTTAAACTCTCAGAACCAC |
| PML812 | AGCGATCCCACCAAATTCAGCTC |
| PML813 | TGAACAACGTGGGCTCCAATCC |
| PML814 | TCGAGTTAAGGCCTGCAGACAG |
| PML815 | TACACAGACTTCGGCCACCTTC |
| PML818 | TCTCGCAACGGGAGAATCACATC |
| PML819 | CCACCGTGGATTTGTCCGTCTTAG |
| PML822 | ATGGGATGTCAACCGGAGTCAGAG |
| PML823 | GCGTCCTGATTAATGCACCAAACC |
| PML839 | CCTTTTGTCTCCCTTTTGTCTCCCTTTTGTCTCCCTTTTGTCTC |
| PML840 | GAGACAAAAGGGAGACAAAAGGGAGACAAAAGGGAGACAAAAGG |
| PML841 | CCTTTTAGGTCCCTTTTAGGTCCCTTTTAGGTCCCTTTTAGGTC |
| PML842 | GACCTAAAAGGGACCTAAAAGGGACCTAAAAGGGACCTAAAAGG |
| PML851 | TCAAAAGGGCTCGATCTCCCATGG |
| PML852 | TCACAACGGTTCTATCTCCCACAATGA |
| PML1068 | ggggaagtggatgagacacaggagatgagacacaatggatgaatg |
| PML1069 | CATTCATCCATTGTGTCTCATCTCCTGTGTCTCATCCACTTCCCC |
| PML1082 | TGTGAGAGAGGGGGGGGGTGTCAGTGTCTCCGGCTTCAGTTTT |
| PML1083 | AAAACTGAAGCCGGAGACACTGACACCCCCCCCCTCTCTCACA |
| PML1084 | GTGTTGTGTGAGAGAGGGGGGGGGCGGCTTCAGTTTTTTTGTG |
| PML1085 | CACAAAAAAACTGAAGCCGCCCCCCCCCTCTCTCACACAACAC |
| PML1094 | GGTGATCAGAGGGGAAGTGGATCAATGGATGAATGAAGATACGAG |
| PML1095 | CTCGTATCTTCATTCATCCATTGATCCACTTCCCCTCTGATCACC |
| PML1108 | AGGGTTTGTCCGGTCTGGGAATACATATATGTTGGAGACATGCTC |
| PML1109 | GAGCATGTCTCCAACATATATGTATTCCCAGACCGGACAAACCCT |
| PML1110 | TGAGGAGGGTTCGGTCTGGGAATACATATATGTTGTGCTCGCAGT |
| PML1111 | ACTGCGAGCACAACATATATGTATTCCCAGACCGAACCCTCCTCA |
| EF1α-F’ | ACGCGTTGTTGGCTTTCACTTTG |
| EF1α-R’ | GTGGTTGCGTCCATCTTGTTGC |
| PpIAA1A-F’ | Atccgggagtccgagcttc |
| PpIAA1A-R’ | ggttctgcgcaggaggtg |
| PpIAA1B-F’ | cggtggtcagaatgggtca |
| PpIAA1B-R’ | Cccacagtctggttctgcg |
| PpIAA2-F’ | tgccttgggactggttcatc |
| PpIAA2-R’ | cacagcaccttgggctttca |
| *PpIAA2* genomic region-F’ | acagcctaggcaatgaagtttagtggtgaaggt |
| *PpIAA2* genomic region-R’ | tctggatcccccacagcaccttgggct |
| dCAPS (*Bsa*BI digestion) | gcagaatcagacagtgggAtgg |
| dCAPS | gcataggcacagatcgacag |
