# Supplementary material for: Constitutive auxin response in Physcomitrella reveals complex interactions between Aux/IAA and ARF proteins
Source: eLife. 2016 Jun 1;5:e13325. doi: 10.7554/eLife.13325 (PMC4889330; doi:10.7554/eLife.13325)
Supplement: Supplementary file 4. — DOI: http://dx.doi.org/10.7554/eLife.13325.021 [file elife-13325-supp4.docx]

Supplementary File 4

| Name | Description | Resistance Gene | Source |
| --- | --- | --- | --- |
| pBNRF | (pBiLox+35S:nptII:camv) | nptII | ([Thelander et al., 2007](#_ENREF_49)) |
| pBNRF-GUS | (pBiLox+35S:nptII-GUS:camv) | nptII | ([Lavy et al., 2012](#_ENREF_30)) |
| pBHRF/ pMP1159 | (pBiLox+2x35S:Hyg:camv) | HygR | <http://labs.biology.ucsd.edu/estelle/moss2.html> (updated version of ([Thelander et al., 2007](#_ENREF_49))) |
| pMP1377 | pUK-Pp108**B**-nptII-Ubi:Gateway:4xMyc:nos | nptII | http://labs.biology.ucsd.edu/estelle/moss2.html |
| pMP1432 | pUK-Pp108**B**-Gent-GmHsp17.3B:Gateway:e9 | Gent | http://labs.biology.ucsd.edu/estelle/moss2.html |
| pUGGi | Hyg-pUbi-GUS inverted repeats:Gateway:nos | HygR | ([Bezanilla et al., 2005](#_ENREF_4)) |
| pUGi | Hyg-pUbi-GUS inverted repeats: nos | HygR | ([Bezanilla et al., 2005](#_ENREF_4)) |
| pTHUBiGate | Pp108  -Hyg-pUBi:Gateway:nos | HygR | ([Vidali et al., 2007](#_ENREF_54)) |
